# Efficacy of a fine fiber film applied with a water‐based lotion to improve dry skin

**DOI:** 10.1111/srt.13149

**Published:** 2022-04-12

**Authors:** Yu Gabe, Masayuki Uchiyama, Shun Sasaoka, Naomi Amari, Asuka Imai, Akira Hachiya, Akira Kiyomine

**Affiliations:** ^1^ Biological Science Research Kao Corporation Odawara Japan; ^2^ Skin Care Products Research Kao Corporation Sumida Japan; ^3^ Analytical Science Research Kao Corporation Sumida Japan; ^4^ Processing Development Research Kao Corporation Haga Japan; ^5^ Kao R&D Kao USA Inc. Cincinnati Ohio USA; ^6^ Planning and Implementation Kao Corporation Sumida Japan

**Keywords:** conductance, direct‐electrospinning (D‐ES), dry skin, fine fiber (FF), hydration

## Abstract

**Background:**

Dry skin can trigger eczema that affects >10% of the US population. Dressing films have been developed to improve diseased skin, but there is limited knowledge about their effects, especially for dry skin‐related symptoms. We developed an electrospinning method that creates a coating film, called a fine fiber (FF) film, characterized by the production of a transparent, thin, flexible, and adherent membrane on the skin surface.

**Objective:**

The aim of this pilot study was to examine the effects of the FF film on dry skin.

**Methods:**

Three treatments (lotion only, lotion with the FF film, and lotion with an alternative film) were designed to treat subjects with rough skin on their lower legs. Twenty‐four females were enrolled and used either a water‐based lotion U or a petrolatum‐based lotion P and the FF film for 2 weeks followed by a regression phase for 1 week. Skin hydration and roughness scores were assessed as were the subjects’ perceptions of the effects.

**Results:**

When the FF film was applied with lotion U, skin hydration was significantly improved even after 1 week, accompanied by a significant improvement of skin roughness and an increase in skin hydration by the end of the regression phase. An evaluation of moisture permeability suggested that the FF film, especially with lotion U, performed as a semipermeable membrane with optimal moisture healing effects on dry skin.

**Conclusion:**

The FF film together with a water‐based lotion is a promising treatment to quickly improve dry skin conditions.

## 1 INTRODUCTION

Dry skin is characterized by dehydration of the stratum corneum, the appearance of a rough and scaly skin surface and an impaired barrier function of the skin, which frequently induces itchiness, and is a common skin problem around the world.^1^, ^2^ Dry skin is also called xerosis, xeroderma, and asteatosis and is known to be a cause of various skin diseases such as dermatitis and eczema.^3^ It has even been pointed out that the formation of wrinkles and spots, which are major manifestations of photoaging, is associated with dry skin.^4^, ^5^ Although many moisturizers (lotions) have been developed to improve dry skin, they have been used as palliative care and have not sufficiently met the diverse needs of consumers, particularly from the viewpoint of their usability and long‐lasting effects, that is, low compliance.^6^, ^7^ On the other hand, occlusive treatments (dressing films and/or tapes) are frequently used to increase the adhesion and efficacy of topical ingredients. Several types of dressing films have been developed especially for wound healing.^8^, ^9^ However, little is known about the application of dressing films for treating dry skin,^10^, ^11^ and it is considered that they are not suitable to cover a wide range of skin areas.

To address these issues, we succeeded in developing a novel film consisting of extra‐fine fibers, called fine fiber (FF), which provides a uniform, thin, and flexible membrane with great adhesion to the skin that can be applied to large skin areas. In the process of creating the FF film, a polymer solution is sprayed onto the skin surface using a portable device with the principle of direct electrospinning (D‐ES),^12^ after which the solvent immediately evaporates so that an FF film is directly created on the skin. While the FF film itself is white, it immediately turns transparent, and it appears as if there is no film on the skin surface when the FF film is applied to the skin together with a lotion. In addition to the physical properties of the FF film related to favorable usability and transparent appearance, it would be more beneficial, especially for those who are concerned about skin dryness‐related symptoms, if the FF film could improve skin conditions in a biological manner.

In the present study, we demonstrated the effects of the FF film together with two types of lotions by comparing them with a commercially available adhesive film.

## 2 MATERIALS AND METHODS

### 2.1 Clinical study design

The study was a within‐subject, two cell design study, consisting of a 3‐day dry‐out phase, a 14‐day treatment phase, and a 7‐day regression period. During the 14‐day treatment phase, each subject was treated with one of the lotions (U or P) on both legs and then applied a transparent adhesive film (Tegaderm 1626W, 3M, St Paul, MN, USA) on one leg and an FF film created using a portable device onto the other leg. Measurements were taken at baseline, at weeks 1 and 2, and at the end of the regression phase for 7 days. Twenty‐four healthy subjects (Caucasian or African‐American females who were between the ages of 31–54 with a mean age of 46.0 years old) were enrolled in this study. Those subjects were divided into two groups who used different lotions, a water‐based lotion U group (group 1, 17 females with a mean age of 45.2 years old), and a petrolatum‐based lotion P group (group 2, seven females with a mean age of 48.0 years old). The formulations of these lotions are shown in Table S1. This study was conducted according to the Declaration of Helsinki principles using a protocol ethically reviewed and approved by the organization Kao USA Inc. Informed consent to participate in this study was obtained from each subject before commencement of the study.

### 2.2 Measurements of skin conditions

Instrumental evaluations were conducted in an air‐conditioned room with a temperature of 21 ± 1°C and a relative humidity of less than 40%. Each subject washed both of her lower legs with a detergent, entered the room, and acclimated for at least 15 min, during which time she completed a questionnaire about the skin appearance and feeling on her lower legs. Three 5 × 5 cm areas were marked on the outer surface of each leg where the skin dryness was similar to each other.

The measurements of skin capacitance and conductance (hydration) were conducted on all test areas using a Corneometer MPA 580 (Courage‐Khazaka electronic GmbH, Köln, Germany) and a Dermalab Moisture Meter (Cortex Technology, Hadsund, Denmark), respectively. Magnified images of each lower leg area were also taken using a Visioscope (Courage‐Khazaka electronic GmbH).

### 2.3 Assessment of dryness score

Observer dryness scores on each lower leg area were assessed by trained experts according to a reference consisting of 5 grades from 0 to 4 with half‐way scores (e.g., 1.5, 2.5) (Figure S1).

### 2.4 Product application

Each subject applied the assigned test product (Lotion U or P) to her right and left lower legs from the knee to the ankle by herself every day during the 14‐day treatment phase ensuring that the lotions were evenly distributed and were fully absorbed. Subsequently, the FF solution was applied using the device (0.10 ml/min) over the area where the lotion had already been applied until the area was completely and evenly covered (60 s).

At the same time, a commercially available adhesive film (Tegaderm) was placed over the assigned areas on the other leg, keeping it as straight as possible to ensure good contact. The FF film and the adhesive film remained on the skin surface for at least 16 h until the subject intentionally removed the products while taking a shower the next morning, after which the lotion and film or membrane was reapplied each day, throughout the 14‐day treatment phase.

### 2.5 Fine fiber network formation on the skin surface

To form a thin and durable FF network‐based film on the skin surface, we focused on the ES method and developed a device that enables the direct ES of FF onto the skin. The ES method is a technique to inject a positively charged polymer solution toward a negatively charged object surface. This device uses an electric field to control the formation and deposition of polymers ideally composed of a single continuous filament in a remarkably efficient and rapid manner. A polymer solution that is discharged from the tip of the nozzle of the device comes out from a discharge port, and while being drawn by an electric field, the solvent evaporates and flies toward the target surface. The formulation of the polymer solution is shown in Table S2.

### 2.6 Moisture permeability

The moisture permeability of the FF film with or without the lotions and the Tegaderm without any lotion was evaluated according to the cup method of Japan Industrial Standards Z0208. Briefly, the FF film with or without lotion or the Tegaderm were mounted on the mouths of cups containing anhydrous calcium chloride. The cups were then sealed and placed in an incubator at a temperature at 25°C and a relative humidity of 90% for 8 h. The moisture permeability of the film is expressed as units of g/m^2^/24 h by applying the weight difference of calcium chloride in the cup before and after the measurement.

### 2.7 Statistics

The level of significance of differences was analyzed by Tukey's test or by the Friedman test (with Bonferroni correction). Differences in mean or raw values among treatment groups were considered significant when *p* < 0.05.

## 3 RESULTS

### 3.1 Improvement of skin roughness and hydration after treatment with the FF film together with a water‐based lotion

**FIGURE 1:**
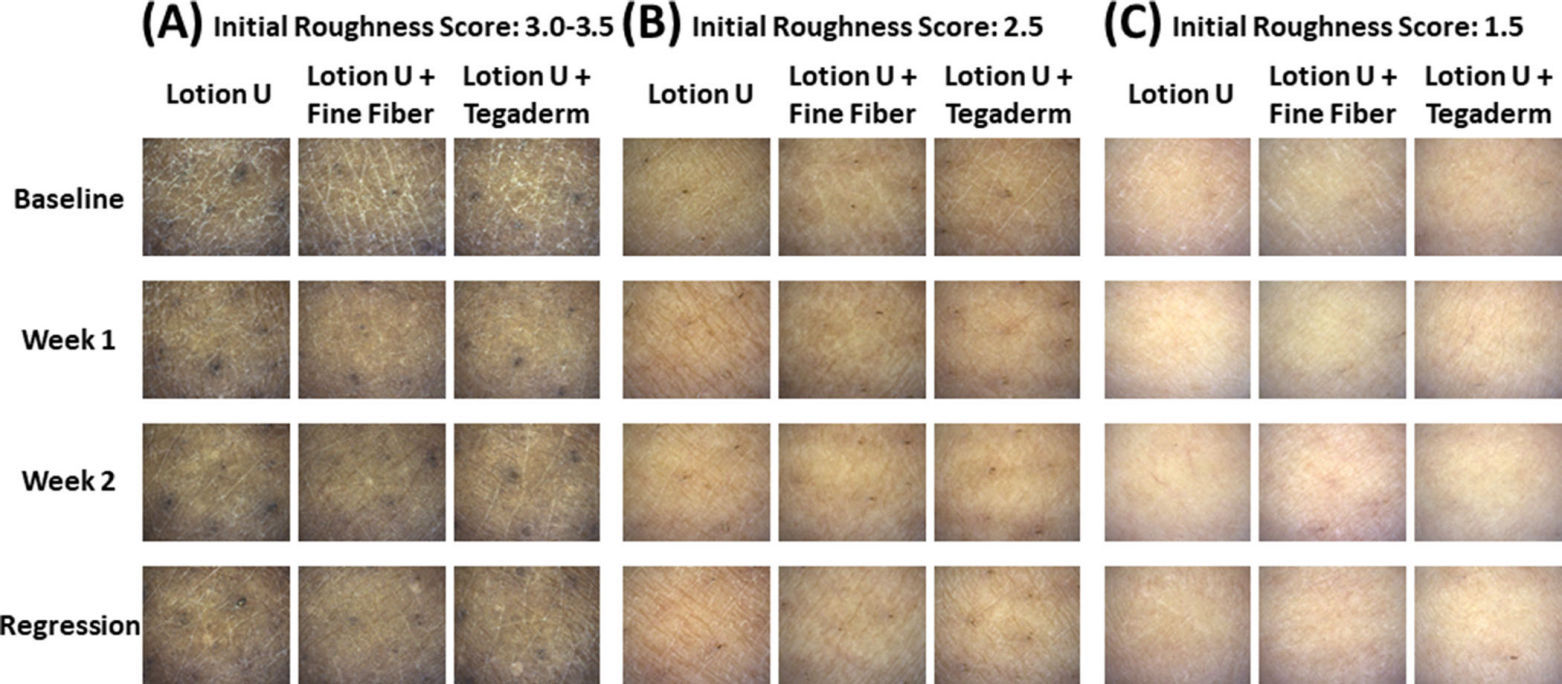
Images of skin after different treatments with lotion U. Magnified images of skin taken at week 1, week 2, and the end of the regression phase for 7 days using a Visioscope. Three types of treatments were carried out on the lower legs (lotion U alone, lotion U with the fine fiber (FF) film and lotion U with an adhesive film). Representative images are shown of subjects with skin with initial roughness scores of 3.0–3.5 (A), 2.5 (B), and 1.5 (C)

**FIGURE 2:**
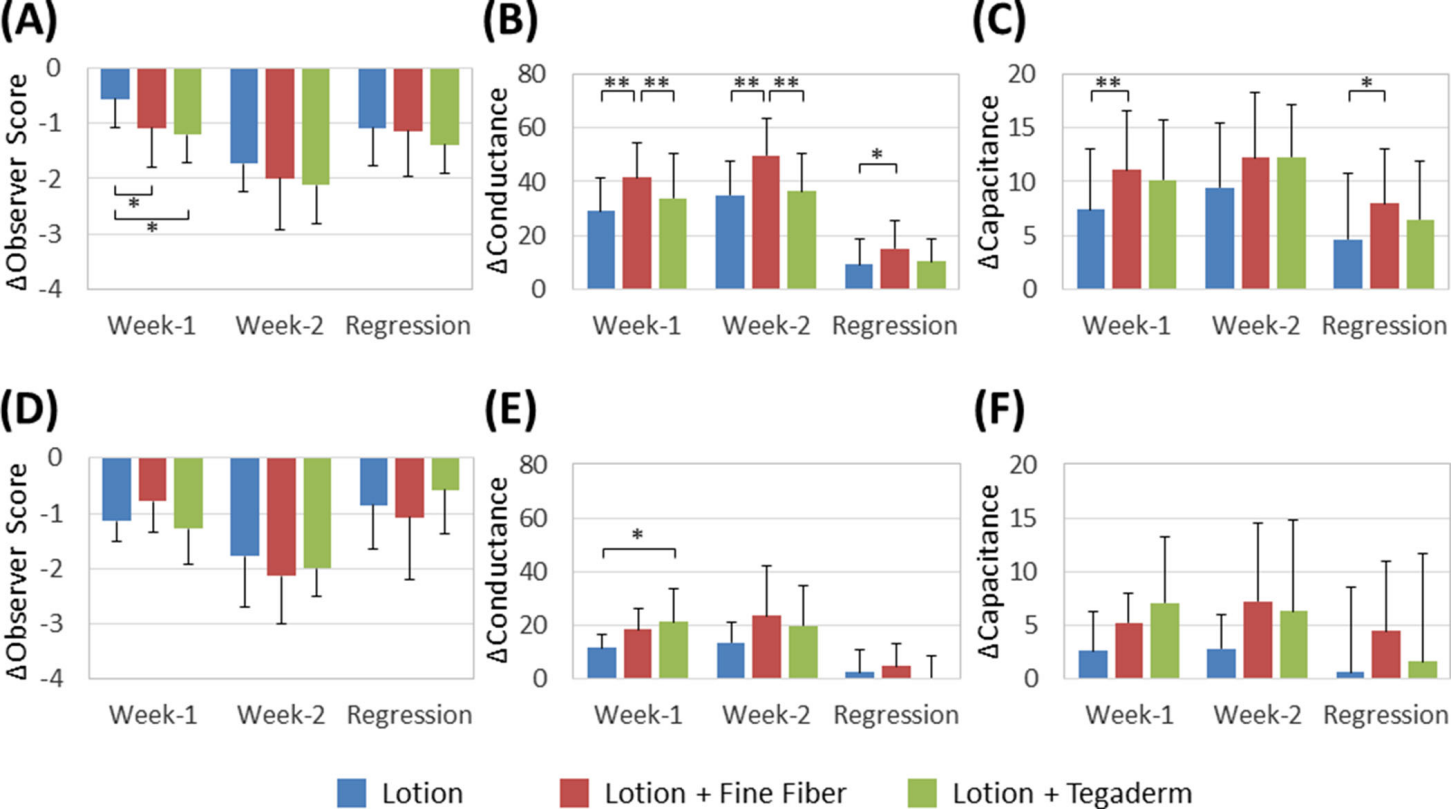
Changes in skin roughness and hydration after the different treatments with lotions U or P. Changes in observer scores (A and D), conductance (B and E) and capacitance (C and F) were measured at week 1, week 2, and at the end of the regression phase for 7 days. Three types of treatments were carried out on the lower legs (lotion alone [blue], lotion with the fine fiber (FF) film [red], and lotion with the adhesive film [green]). Seventeen females (group 1) used lotion U (A, B, and C), and the other seven females (group 2) used lotion P (D, E, and F). Results are expressed as means ± SDs (*p < 0.05, **p < 0.01 by Tukey's test or by the Friedman test [with Bonferroni correction])

In the present study, we recruited a total of 24 subjects who suffered from dry skin on their lower legs according to the grades of skin roughness ranging from 0 to 4 (grade 4 represents the severest roughness). The subjects were then divided into two groups with comparable scores of initial skin roughness who used different lotions (group 1 used a water‐based lotion U, while group 2 used a petrolatum‐based lotion P). Three types of treatment remedies (water‐based lotion U only, FF film with lotion U, and a commercially available adhesive film with lotion U) were provided to group 1 composed of 17 subjects for 2 weeks. The skin on their lower legs was evaluated before the start of treatment, after the applications at 1 and 2 weeks and at the end of the 1 week regression phase. The initial roughness scores of each area treated with lotion U only, with the FF film with lotion U or with the adhesive film with lotion U were 2.5 ± 0.8, 2.4 ± 0.9, or 2.6 ± 0.8 (means ± standard deviations [SDs]), respectively. A significant improvement in appearance and roughness score of skin treated with the FF film together with lotion U occurred even at week 1 compared to the treatment with lotion U only (Figures 1 and 2A). When applying the commercially available adhesive film with lotion U, a significant improvement in roughness score was also observed at week 1 (Figure 2A). On the other hand, skin hydration, reflected by the conductance value, was significantly increased in areas treated with the FF film with lotion U at weeks 1 and 2 compared to the application of lotion U only to or the adhesive film with lotion U. The significant increase of conductance at areas covered by the FF film with lotion U was maintained until the regression measurement (Figure 2B). Regarding the skin hydration, represented by capacitance, a significant increase was detected in areas treated with the FF film with lotion U at week 1 and at the end of the regression phase, while no significant changes were detected at areas treated with the commercially available adhesive film compared to treatment with lotion U only (Figure 2C).

### 3.2 Perceptions of skin improvement after treatment with the FF film combined with the water‐based lotion U

**FIGURE 3:**
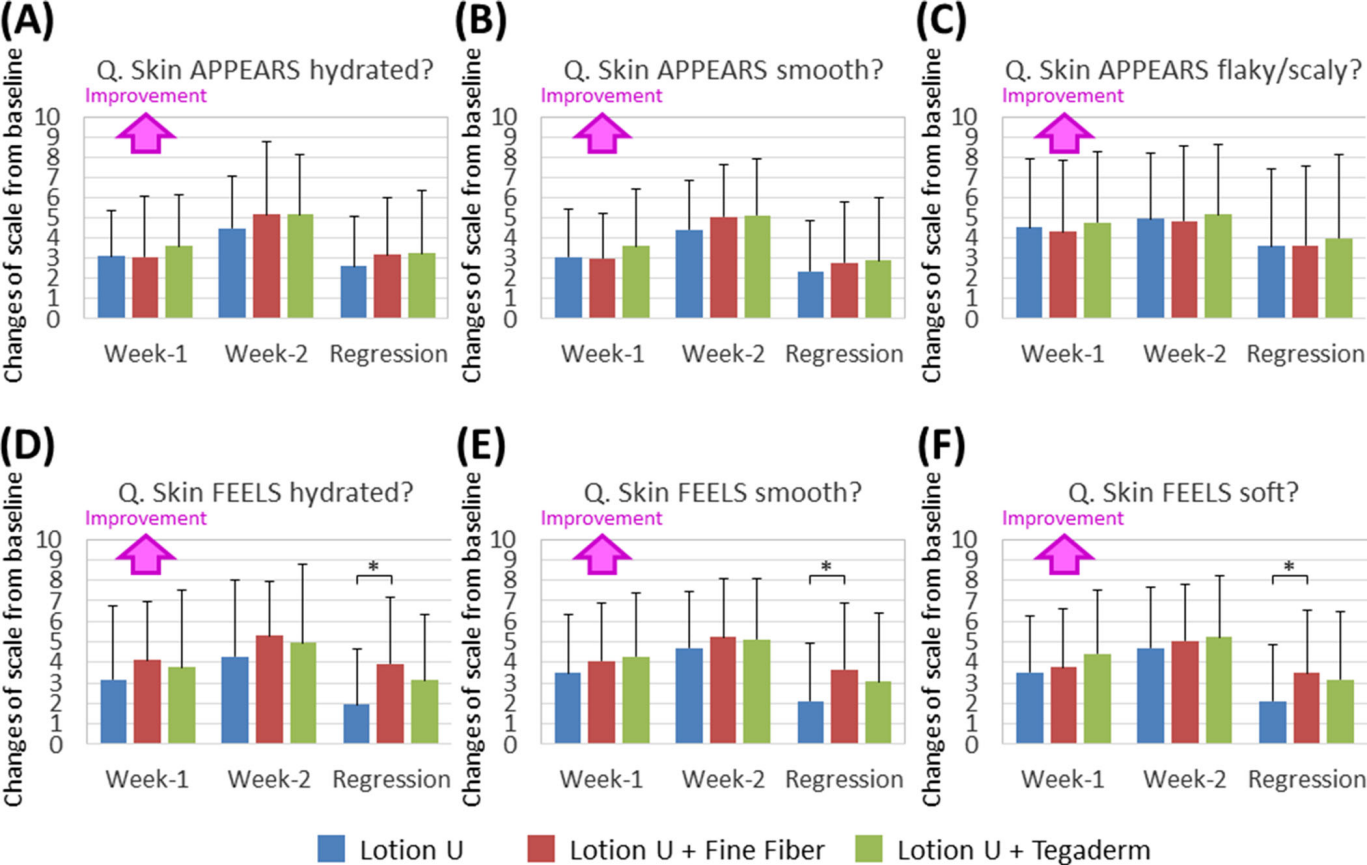
Changes in appearance and feeling of the skin perceived by the subjects after the different treatments with lotion U. Changes in skin appearance (A, B, and C) and feeling (D, E, and F) were detected at week 1, week 2, and at the end of the regression phase for 7 days by a questionnaire. Three types of treatment were carried out on the lower legs (lotion U alone [blue], lotion U with the fine fiber (FF) film [red], and lotion U with an adhesive film [green]). Results are expressed as means ± SDs (*n* = 17) (*p < 0.05 by the Friedman test [with Bonferroni correction])

In parallel with the measurements of skin conditions, a questionnaire survey was performed. Scores in skin appearance and skin feelings before and after the product usage were evaluated using a 10‐point level by subjects’ self‐evaluation. Although significant differences related to perceptions of skin appearance among the three treatments were not found, the feeling perception of skin improvement related to hydration, smoothness, and softness after treatment with the FF film with lotion U was significantly higher at the end of the regression phase compared to treatment with the lotion U only (Figure 3), indicating that a long‐lasting effect perceived at least by feeling is provided by the FF technology.

### 3.3 Slight improvement of dry skin‐related symptoms after treatment with the FF film combined with the petrolatum‐based lotion P

To clarify the compatibility of lotions with the FF film, seven other subjects (group 2) with comparable dry skin conditions to group 1 received the treatment with lotion P instead of lotion U on their lower legs in combination with the FF film or the commercially available adhesive film according to the same protocol used for group 1. Both lotion U and lotion P are oil in water type emulsions, and lotion P is more viscous than lotion U because of its greater content of petrolatum. The initial roughness scores on each area treated with lotion P only, with the FF film with lotion P or with the commercially available adhesive film with lotion P, were 2.5 ± 0.8, 2.4 ± 0.7, or 2.2 ± 0.6 (means ± SDs), respectively. Even though the roughness score at week 1 was improved at all three areas compared to the initial value before usage, there were no significant differences among the three treatments (Figure 2D). Regarding the skin hydration represented by capacitance and conductance, some increases were observed in areas treated with the FF film with lotion P or with the commercially available adhesive film with lotion P compared to lotion P only despite the smaller increases than those obtained with lotion U (Figure 2E,F). In parallel, the questionnaire indicated that there were no remarkable differences among the three treatments in group 2 subjects regarding either the skin appearance or feelings (Figure S2).

### 3.4 Semipermeable property of the FF film combined with lotion U or P

**FIGURE 4:**
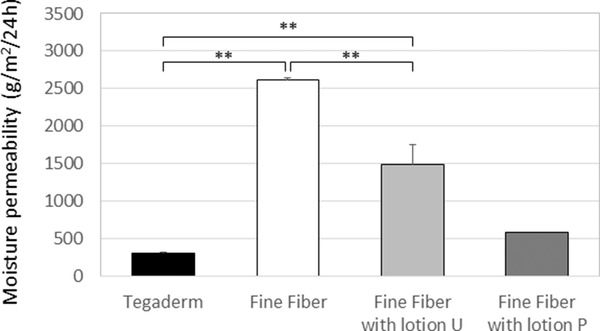
Moisture permeability of the fine fiber (FF) film with or without lotions and the Tegaderm. The moisture permeability of the FF film alone (white), the FF film with lotion U (light grey), the FF film with lotion P (dark grey), and the Tegaderm alone (black) was evaluated at a temperature of 25°C and a relative humidity of 90% according to the cup method of Japan Industrial Standards (JIS) Z0208. Results are expressed as means ± SDs (*n* = 3) except for the FF film with lotion P (*n* = 2) (**p < 0.01 by Tukey's test)

Since the different efficacies of several treatment remedies on dry skin in this study were considered to characterize the properties of the FF film when applied together with lotion U or lotion P, we evaluated the moisture permeability of the FF film with or without lotions. The moisture permeabilities of the FF film with lotion U and with lotion P were 1489 and 589 g/m^2^/24h, respectively, which were significantly lower than the FF film alone (2618 g/m^2^/24h) and were significantly higher than the Tegaderm alone (313 g/m^2^/24 h) (Figure 4). Comparing lotion U with lotion P, the moisture permeability of the FF film with lotion U was approximately 2.5 times higher than the FF film with lotion P. These results indicate that the FF film with lotions performs as a semipermeable membrane and its moisture permeability can be changed by the accompanied products.

## 4 DISCUSSION

Occlusive treatments such as dressing films or tapes have been widely applied to wound areas mainly to accelerate the healing process and to prevent infections.^8^, ^9^, ^13^ Those films are frequently used for treating atopic dermatitis and eczema,^14^ which are closely related to dry skin symptoms. However, their proactive introduction to dry skin‐related treatments has been rarely discussed and has been considered to be difficult to apply to large skin areas.^15^ In addition, little is known about what kind of films would be more beneficial to improve dry skin conditions to our knowledge.^10^ To help subjects who are suffering from or are concerned about dry skin‐related conditions, the D‐ES method was developed to directly create a transparent, thin, flexible, and comfortable adherent FF film on a wide range and/or variety of skin areas. Since the FF film consists of microfibers, we thought that it would perform as a semipermeable membrane that could substantially absorb and sustain the release of liquids due to its capillarity. A comparison of the physical properties between the FF film and commercially available transparent adhesive films (Table S3) prompted us to carry out a pilot study to determine the benefits of the FF film together with water‐based or petrolatum‐based lotions. The water‐based lotion U contained 21% glycerin with a high moisturizing effect and the petrolatum‐based lotion P contained 75% petrolatum white with a high occlusion effect. These unique properties of the FF film led us to hypothesize that the FF film could maximize the effect of lotions from the viewpoint of dry skin restoration.

It would be of interest to validate our hypothesis by performing a pilot study with appropriate subjects with dry skin symptoms. As we expected, in the case of treating skin with an FF film together with water‐based lotion U, significant improvements of skin roughness and hydration even at week 1 were found compared to the treatment with lotion U alone. Surprisingly, skin hydration represented by conductance was significantly and constantly increased after use of the FF film with lotion U compared to the use of lotion U alone until the regression measurement (Figure 2B). Since the conductance value reflects the water condition of the skin surface layer,^16^ the FF film might affect the function and/or structure of the stratum corneum layer close to the skin surface. On the other hand, the Tegaderm did not show any constant skin hydration effects regardless of whether lotion U or lotion P was used. To clarify the reasons behind such differences between the FF film and the Tegaderm, we focused on their permeability (occlusive properties). The moisture permeability of the FF film with lotion U was 1489 g/m^2^/24 h, which was significantly lower than the FF film alone (2618 g/m^2^/24 h) and was significantly higher than the Tegaderm (313 g/m^2^/24 h) (Figure 4). In addition to reports that complete occlusion of the skin is not a problem if applied temporarily, semi‐occlusion is better for the skin,^17^, ^18^ a semipermeable membrane was also reported to reduce wound contraction and enhance cell migration and reepithelization without irritation.^19^ Rather than the general advantages of semi‐occlusive properties toward wound healing, previous studies have suggested the appropriate moisture permeabilities of occlusive or semi‐occlusive membranes. For example, Xu et al. reported that a dressing with a medium permeability of 2028 g/m^2^/24 h maintained an optimal moisture content for wound healing by performing in vitro and in vivo studies.^20^ Furthermore, Visscher et al. also suggested that a semipermeable membrane with 1596 g/m^2^/24 h brought a high water‐holding capacity and barrier repair rate following tape stripping.^21^ Taken together, from the viewpoint of evaluating moisture permeability, it would make sense to consider that the combination of a semi‐occlusive FF film with lotion U might provide a proper range of healthy water‐holding capability to restore the barrier function of the skin even within 1 week.

In parallel, the consideration of the compatibility of lotions with the FF film might provide some insight behind the differences among the remedies discussed above. Whereas the petrolatum‐based lotion P itself seemed to have a higher efficacy to improve roughness score (−1.1 ± 0.4) compared with lotion U alone (−0.6 ± 0.5) at week 1 (Figure 2A,D, *p* = 0.008), the FF film did not improve any skin roughness or hydration scores of lotion P until the end of the study. In terms of the moisture permeability of the FF film with lotion P, it was calculated to be 589 g/m^2^/24 h, which is out of the range of an appropriate moisture permeability to alleviate skin dryness (Figure 4). On the other hand, a booster effect of the FF film with lotion U was observed as expected in the significant skin hydration improvement even at the regression phase, emphasizing a substantial biological action of the combination of the FF film and lotion U to restore the cutaneous barrier function with a long‐lasting effect. Consistently, this restoration was practically perceived by the subjects from the viewpoints of skin hydration, smoothness, and softness as reflected in the questionnaire (Figure 2). This finding led us to consider the mechanism behind the different effects of the two lotions when used with the FF film. The microstructure of FF films indicated by microscopic images clarified the contrasting compatibilities with the FF film. Lotion U was found not to interfere with the clear fiber networks of the FF film that would allow the ingredients of lotion U to uniformly spread on them, even though lotion P actually disturbed the networks and existed in the film in a heterogeneous manner (Figure S3). This observation suggested that some compatibility of lotions is required for the FF film to keep its proper network that might significantly affect the moisture permeability that is essential for the recovery of dry skin‐related symptoms.

In summary, our pilot study demonstrated that the application of the FF film together with a water‐based lotion improved dry skin symptoms even in a short period of time. Although additional studies with an increased number of subjects are needed to verify the effects and optimal application conditions of the FF film, treatment with the FF film is considered to be a promising skin care and/or remedy especially for dry skin‐related diseases such as eczema and atopic dermatitis. Our findings not only provide new insights for a fundamental understanding of the incorporation of FF technology into treatments for the aforementioned symptoms but also suggest new strategies to explore materials for both cosmetic and therapeutic applications toward skin dryness refinement.

## CONFLICT OF INTEREST

The authors declare that there is no conflict of interest that could be perceived as prejudicing the impartiality of the research reported.

## Supporting information

Supporting Information
