# Supplementary material for: Efficacy of a fine fiber film applied with a water‐based lotion to improve dry skin
Source: Skin Res Technol. 2022 Apr 12;28(3):465–71. doi: 10.1111/srt.13149 (PMC9907623; doi:10.1111/srt.13149)
Supplement: Supplementary file 1 — Supporting Information [file SRT-28-465-s001.docx]

**SUPPORTING INFORMATION – TABLE AND FIGURE LEGENDS**

**TABLE S1.** Formulations of lotions U and P.

| **Ingredients** | **Contents (% wt/wt)** |
| --- | --- |
| **Lotion U** |  |
| Water | 66.19 |
| Glycerin | 21.00 |
| Methylparaben | 0.30 |
| Cetearyl Alcohol | 3.54 |
| Petrolatum White | 3.75 |
| Behentrimonium Chloride | 1.87 |
| Isopropyl Palmitate | 1.10 |
| Cetyl-PG Hydroxyethyl Palmitamide | 0.50 |
| Dimethicone (10 cst) | 1.75 |
| **Lotion P** |  |
| Petrolatum White | 75.0 |
| Cetyl Alcohol | 1.8 |
| Stearyl Alcohol | 1.2 |
| Glycerin | 3.0 |
| Phenoxyethanol | 0.3 |
| PEG-60 Hydrogenated Castor Oil | 0.4 |
| Butylene Glycol | 1.0 |
| Water | 17.3 |

**TABLE S2.** Formulation of the polymer solution for the FF film.

| **Ingredients** | **Contents (% wt/wt)** |
| --- | --- |
| Poly vinyl butyral | 11.0 |
| Phytosteryl/Octyldodecyl Lauroyl Glutamate | 4.6 |
| Ethanol | 84.0 |
| Water | 0.4 |

**TABLE S3.** Air permeability of the FF film and commercialized transparent adhesive films.

| **Films** | **Gurley air permeability (s)** |
| --- | --- |
| Fine Fiber film | 0.3 |
| Tegaderm™ 1626W (3M, St Paul, MN, USA) | >300 |
| DERMALIZE PRO (D-LIZE srl, Grado, Italy) | >300 |
| SANIDERM (Saniderm Medical LLC, Lehi, UT, USA) | >300 |

Air permeability of the films was quantified using a Gurley tester (No.323-AUTO GURLEY TYPE DENSOMETER, YASUDA SEIKI SEISAKUSHO, LTD., Hyogo, Japan) according to ISO 5636-5 (2013).


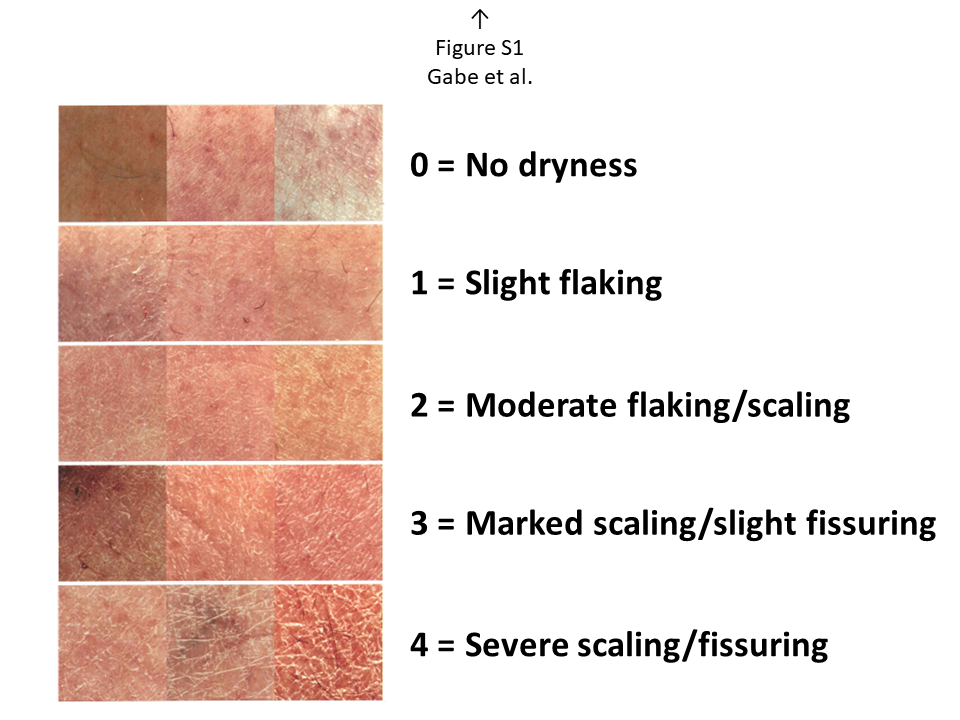


**FIGURE S1.** Observer dryness scoring.

Observer dryness scoring was assessed using this rating scale. Half scores were permitted.


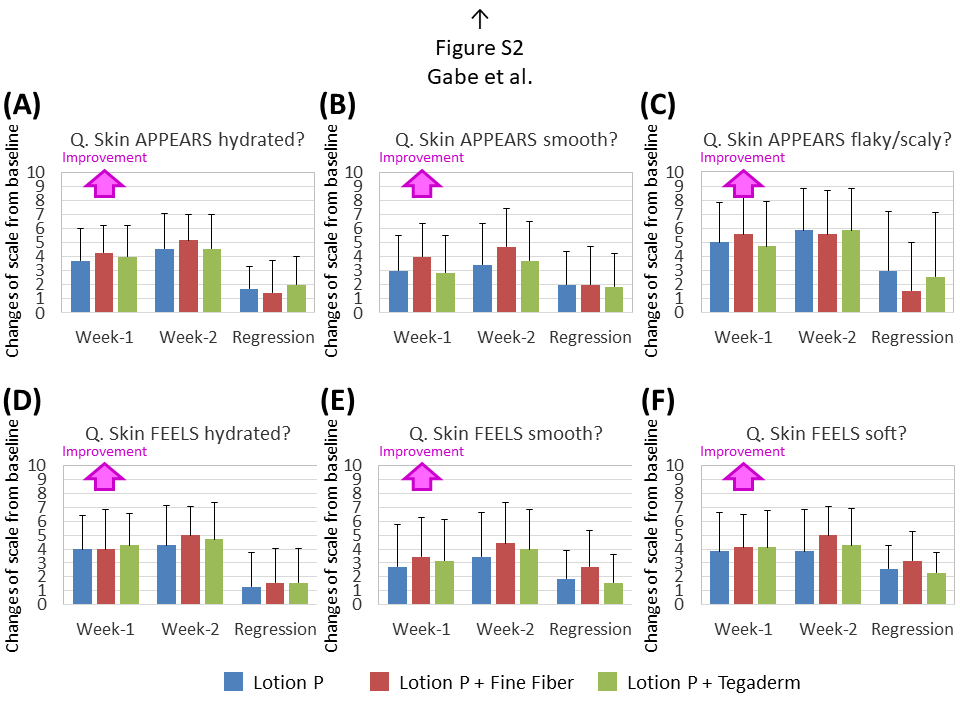


**FIGURE S2.** Changes in appearance and feeling of the skin perceived by the subjects after the different treatments with lotion P.

Changes in skin appearance (A, B and C) and feeling (D, E and F) were detected at week 1, week 2 and at the end of the regression phase for 7 days by the questionnaire. Three types of treatments were carried out on the lower legs (lotion P alone (blue), lotion P with the FF film (red) and lotion P with an adhesive film (green)). Results are expressed as means ± SDs (n = 7).


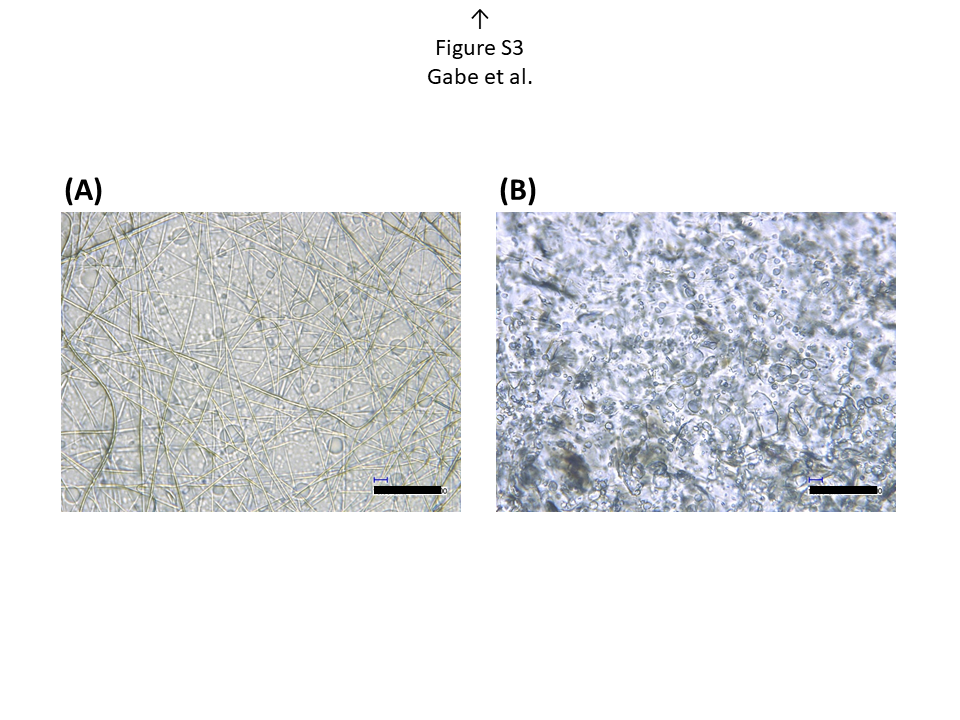


**FIGURE S3.** Images of the FF film applied with the lotions.

The FF film with lotion U (A) or lotion P (B) was developed on a glass slide. Magnified images were taken using a microscope (Hirox Co., Ltd., Tokyo, Japan). Scale bars represent 50 µm.
